# Supplementary material for: Knowledge, Attitudes, and Practices Regarding Air Pollution among Medical Students
Source: Int J Environ Res Public Health. 2024 Jun 17;21(6):789. doi: 10.3390/ijerph21060789 (PMC11204335; doi:10.3390/ijerph21060789)
Supplement: Supplementary file 1 [file ijerph-21-00789-s001.zip › ijerph-2937465-supplementary.pdf]

SUPPLEMENTARY MATERIAL: SURVEY

1. SOCIODEMOGRAPHIC DATA AND PERCEPTIONS

**Gender**	Female
	Male
**Age**	
**Semester**	1
	2
	3
	4
	5
	6
	7
	8
	9
	10
	11
	12

**Monthly Family Income**	Less than one minimum wage
	Between 1 and 2 minimum wages
	Between 3 and 4 minimum wages
	More than 4 minimum wages
**Perception of Education Received on Health and Air Quality Relationship**	Not received
	Excellent
	Good
	Regular
	Poor
**Are you satisfied with the city's air quality?**	No
	Yes
**Perception of Air Quality in the City (Previous Year)**	Excellent
	Good
	Regular
	Poor
**Perception of Current Air Quality in the City**	Excellent
	Good

	Regular
	Poor
**Considers that air quality in the city will improve in the coming years**	No
	Yes

2. KNOWLEDGE QUESTIONNAIRE

QUESTION	Completely Disagree	Disagree	Agree	Completely Agree
Exposure to air pollution increases the risk of respiratory diseases.				
Exposure to air pollution increases the risk of cardiovascular diseases.				
Exposure to air pollution increases the risk of neurological diseases.				
Exposure to air pollution increases the risk of inflammatory skin diseases.				
Exposure to air pollution increases the risk of complications in pregnancy and the fetus.				
Vehicles using fossil fuels contribute to air pollution.				
Livestock farming contributes to air pollution.				
Deforestation contributes to air pollution.				
Household cleaning products in aerosol contribute to air pollution.				
Personal hygiene products in aerosol contribute to air pollution.				
Staying hydrated by drinking at least 1.5 liters of water per day reduces the harmful effects of air pollution.				
Eating healthily reduces the harmful effects of air pollution.				
Having plants (indoor) in the home protects against air pollution.				
Using face masks protects against air pollution.				
Avoiding outdoor exercise during rush hour reduces exposure to polluted air.				

3. ATTITUDES QUESTIONNAIRE

QUESTION	Completely Disagree	Disagree	Agree	Completely Agree
I am interested in learning/training about air pollution.				
Health professionals should inform their patients about the effects of air pollution.				
Environmental health is a topic that should be addressed in the curriculum of your career.				
Air pollution is a problem that affects global public health.				
Air pollution is a problem that affects public health in my country.				
Air pollution is a problem that affects public health in my city.				
I would avoid personal hygiene products in aerosol form to protect air quality.				
I would report to authorities if I ever detect irregular emissions of pollutants from an industry.				
I would reduce my regular consumption of dairy products to protect air quality.				
I would reduce my regular consumption of beef to protect air quality.				
I would use a bicycle to protect air quality.				
Air pollution is a multifactorial problem; my actions have no impact.				
The issue of air pollution is being overrated; it receives more attention than necessary.				

4. PRACTICES QUESTIONNAIRE

QUESTION	Never	Almost never	Almost always	Always
You consume beef more than 2 times a week.				
You consume dairy products more than 3 times a week.				
You have consulted or inquired about the health effects of air pollution.				
You have plants in your home to purify the air.				
You have consulted or inquired about the main causes or sources of air pollution.				
You have recommended measures to your family or friends to protect themselves from the effects of air pollution.				
You have taken measures to mitigate the health effects of air pollution.				
You have taken measures to protect yourself from air pollution.				
You use masks to protect yourself from air pollution.				
You use a bicycle or other means of transportation that do not require fossil fuels.				
You monitor the air pollution indicators in the municipality where you reside.				
You use aerosol personal hygiene products.				
Aerosol products are used for cleaning your home.				
You do not take action to reduce environmental pollution because others do not either.				
